# Genes influence facial attractiveness through intricate biological relationships

**DOI:** 10.1371/journal.pgen.1008030

**Published:** 2019-04-04

**Authors:** Julie D. White, David A. Puts

**Affiliations:** 1 Department of Anthropology, Pennsylvania State University, University Park, Pennsylvania, United States of America; 2 Center for Brain, Behavior, and Cognition, Pennsylvania State University, University Park, Pennsylvania, United States of America; 3 Center for Human Evolution and Diversity, Pennsylvania State University, University Park, Pennsylvania, United States of America; University of Pittsburgh School of Dental Medicine, UNITED STATES

In Greek mythology, Helen of Troy was so beautiful that her face “launched a thousand ships,” compelling King Menelaus to wage war to reclaim her from Prince Paris. Human preoccupations with beauty are enduring and now support a multibillion-dollar industry. Each day, our brains identify and catalog innumerable datapoints that bear on our impressions of beauty—those related to youth, health, adiposity, complexion, coloration, averageness, symmetry, masculinity/femininity, and personality, to name some of the best characterized (Fig 1) [1]. Congruent with the common saying that “beauty is in the eye of the beholder,” perceptions of attractiveness vary within and among individuals and across cultures. Yet when multiple individuals compare the same set of faces, clear agreement exists both within and between cultures about which faces are most attractive.

**Fig 1:**
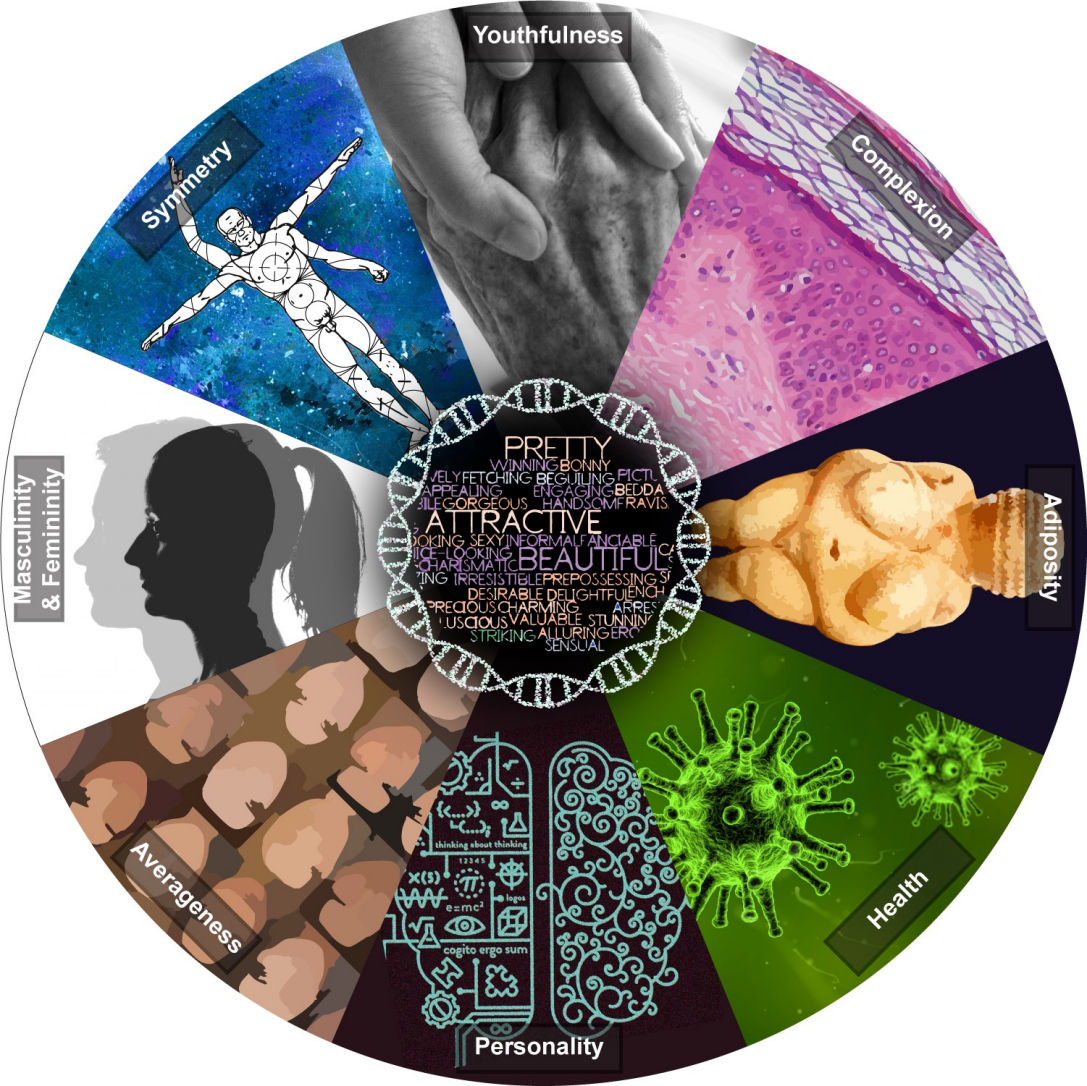
Some well-characterized features influencing perceptions of facial attractiveness. Depicted are physical traits that influence facial attractiveness through changes in shape (e.g., adiposity, averageness, masculinity/femininity, and symmetry) or color (e.g., complexion), as well as qualities influencing facial attractiveness that might also be inferred from both shape and color information (e.g., youthfulness, health, and personality). Relationships between each of these features and attractiveness are likely mediated by a host of biological factors influenced by genotype.

Evolutionary hypotheses concerning the importance of attractiveness and its component factors in mate choice have revolved around the utility of these components in predicting the qualities of prospective mates [1]. For example, preferences for youthful appearance in female faces may function to direct courtship efforts toward those with high reproductive potential. Complexion and adiposity may reflect current health [2,3]. Other traits are purported to represent cues of underlying genes that increase the survival and reproduction of offspring by, for example, providing pathogen resistance, attractiveness, or dominance. Symmetry, masculinity, weight, and averageness have each been linked with indicators of genetic quality [3–5], though many of these relationships are contested [6–8].

## Genetics of facial attractiveness

Given the importance of attractiveness across interpersonal contexts, studies that investigate the underlying genetics of facial attractiveness, such as the one reported by Hu and colleagues [9] in this issue, are invaluable but should be interpreted carefully, commensurate with the complexity of attractiveness as a phenotype. Although Hu and colleagues report considerably lower heritability estimates for facial attractiveness than a previous estimate [10], perhaps due to modest interrater reliability (S13 Fig in [9]), evidence of heritability suggests that searches for underlying loci associated with attractiveness may bear fruit. Datasets with genome-wide genetic data and rated facial attractiveness are rare and time-consuming to gather, and Hu and colleagues smartly leverage a large, pre-existing dataset. After testing 6 overlapping sets of facial attractiveness ratings, they find 1 SNP associated with rated facial attractiveness at a study-wide threshold, 1 SNP significant at genome-wide threshold, and 10 suggestively significant SNPs.

Through a series of enrichment tests, Hu and colleagues identify several correlations between attractiveness ratings and genes influencing other traits—namely, body mass index in females and lipid traits in males (Fig 4 in [9]). Indeed, this study manifests as an illustration of the ability of a large GWAS on a complex phenotype to identify genes related to its simpler component traits and correlates. The candidate genes identified for both significant results and nearly all suggestive results have entries in GWAS Catalog for traits related to attractiveness, including skin pigmentation and melanoma, body mass index (BMI), and the BMI-related phenotypes of height and waist–hip ratio (Table 1). Homogeneous skin coloration [11] and red and yellow tints [12] increase ratings of attractiveness cross-culturally, potentially due to the connection between these traits and perceptions of health and youth. The relationship between weight and attractiveness is demographically variable; for example, American men of European descent rate lower weights as more attractive, except in extremely low BMI ranges [13], whereas African American men are more likely to prefer heavier figures [14]. Hu and colleagues also identify candidate genes related to attractiveness that have been previously associated with facial morphology, possibly implicating facial traits (such as those contributing to youthful facial appearance) in perceptions of attractiveness [15]. One explanation for Hu and colleagues finding a genetic association between male-rated female attractiveness and BMI is a mediating relationship whereby the candidate gene (*CDC42EP3*) affects height, which directly influences BMI. Similarly, the genetic association between female-rated male attractiveness and lipid levels observed by Hu and colleagues could be explained by the previously identified impact of candidate genes *CERS2* and *ANXA9* on both high- and low-density lipoprotein cholesterol levels (Table 1). It is also possible that Hu and colleagues find different loci for male- and female-rated attractiveness because men and women seem to vary in the specific traits they perceive as attractive [16].

**Table 1:** GWAS results related to attractiveness. Associations between genes identified in [9] and prior results related to attractiveness, found by searching GWAS Catalog (https://www.ebi.ac.uk/gwas/) for the candidate genes identified in [9] and selecting those results related to morphology, traits that influence attractiveness ratings, and the lipid traits described in [9].

Hu and colleagues		GWAS Catalog				
Trait	Candidate Gene	Trait	SNP	Candidate Gene	P-value	Study accession
MC-AS	*LRP1B*	Aging	rs12474609	*LRP1B*	6.00 x 10^-9^	GCST000378
		Age at menarche	rs12472911	*LRP1B*	2.00 x 10^-7^	GCST000880
						GCST002541
		Body mass index	rs12617004	*LRP1B*	6.00 x 10^-9^	GCST004904
	*PTPRT*	Facial morphology (factor 20)	rs2867028	*PTPRT*	4.00 x 10^-6^	GCST004324
		Eye morphology (Left eye angle of en-ps-ex)	rs6016745	*PTPRT*	6.00 x 10^-6^	GCST006105
		Obese body mass index	rs7263077	*PTPRT*	6.00 x 10^-6^	GCST002828
FC-AS	*LY86*	Obese body mass index	rs4246076	*LY86*, *LY86-AS1*	6.00 x 10^-6^	GCST002829
		Waist-hip ratio	rs1294421	*LOC101928004*	7.00 x 10^-14^	GCST004064
						GCST000829
						GCST001954
	***ANTXRLP1***	Melanin index	rs111256285	*ANTXRLP1*	8.61 x 10^-6^	GCST004219
MC-FS	***CDC42EP3***	Facial morphology (factor 5, width of mouth relative to central midface)	rs116711337	*LOC107985870*	4.00 x 10^-6^	GCST004309
		Height	rs17511102	*LOC105374465*	2.00 x 10^-18^	GCST000817
						GCST001956
	*SPON1*	Facial morphology (factor 1, breadth of lateral portion of upper face)	rs79756450	*LOC101928132*, *SPON1*	6.00 x 10^-7^	GCST004328
FC-FS	*MED30*, *EXT1*	Obese body mass index status	rs3115775	*LOC105375721*	8.00 x 10^-6^	GCST002828
		Height	rs1198912	*EXT1*	6.00 x 10^-6^	GCST000522
		Cortisol secretion	rs7459527	*EXT1*	2.00 x 10^-6^	GCST001762
	*NXN*	Facial morphology (factor 15, philtrum width)	rs3851779	*NXN*	4.00 x 10^-6^	GCST004319
		Mean arterial pressure	rs747685	*NXN*	6.00 x 10^-7^	GCST002497
		Diastolic blood pressure	rs747687	*NXN*	2.00 x 10^-7^	GCST002497
MC-MS	*RAB11FIP4*	-	-	*-*	-	-
FC-MS	*CERS2*, *ANXA9*	High density lipoprotein cholesterol measurement	rs267738	*CERS2*	6.00 x 10^-12^	GCST006611
		Low density lipoprotein cholesterol measurement	rs267733	*ANXA9*	4.00 x 10^-8^	GCST004233
						GCST002222
		Melanoma	rs1722784	*ANXA9*	2.00 x 10^-6^	GCST001245
	*LOC285692*	-	-	*-*	-	-
	*PDZRN4*, *GXYLT1*	Height	rs1405552	*PDZRN4*	1.00 x 10^-10^	GCST005951
						GCST006368
		Skin pigmentation	rs1902910	*PDZRN4*	2.00 x 10^-6^	GCST004219
		Height	rs285575	*PDZRN4*	7.00 x 10^-8^	GCST002783
		Overweight body mass index	rs11180992	*PDZRN4*	3.00 x 10^-6^	GCST002829
		Height	rs11181001	*PDZRN4*	4.00 x 10^-10^	GCST005951
						GCST006368
		Diastolic blood pressure	rs7965392	*GXYLT1*, *YAF2*	4.00 x 10^-10^	GCST006627

Note: Candidate genes with significant results in [9] are bolded. P-value refers to the P-value in the GWAS catalog study, represented by the GWAS catalog study accession number. AS, all samples; FC, female coders; FS, female samples; GWAS, genome-wide association study; MC, male coders; MS, male samples.

## The future

The results of this study point to underlying genetic architecture mediating attractiveness. In the future, careful multivariate studies testing the relative contribution of each associated locus to the component traits of attractiveness, and to attractiveness corrected for those traits, will help researchers unravel and interpret the genetic architecture of this important and complex phenotype. Of course, replication in the few other datasets possessing both genotype and attractiveness data will aid in validation and resolution of these results, and sequencing studies will help clarify the possibly functional variants at each locus and further explore their effect on attractiveness or its related components. Hu and colleagues also briefly mention signatures of selection on alleles associated with male facial attractiveness. This result is especially intriguing and brings up several avenues for future research. Do other secondary sex traits, such as vocal characteristics, show similar signatures of selection in males, indicating sexual selection among our male ancestors [17]? Importantly, does the selection pressure driving the strong relationship between allele frequency and male attractiveness reflect pressure upon the attractiveness per se, or upon related phenotypes, such as lipid metabolism? How do potential signatures of selection fit in with previous evolutionary hypotheses? If there are causative pathways between the associated loci and attractiveness, have cross-cultural variations in preference [18] led to population-specific allele variation at these candidate attractiveness loci?

When contemplating how to depict Helen of Troy, the 5th century BC painter Zeuxis recognized the challenge of identifying the features that define beauty [19]. This challenge remains, and understanding the biological factors that influence attractiveness is equally compelling and complex. Hu and colleagues bring forth a valuable initial foray into the genetic architecture of attractiveness and emphasize the intricate relationships between attractiveness and other visible traits.

## References

[1] Little AC, Jones BC, DeBruine LM. Facial attractiveness: evolutionary based research. Philos Trans R Soc Lond B Biol Sci. 2011;366: 1638–59. 10.1098/rstb.2010.0404 21536551 PMC3130383

[2] Stephen ID, Law Smith MJ, Stirrat MR, Perrett DI. Facial Skin Coloration Affects Perceived Health of Human Faces. Int J Primatol. 2009;30: 845–857. 10.1007/s10764-009-9380-z 19946602 PMC2780675

[3] de Jager S, Coetzee N, Coetzee V. Facial Adiposity, Attractiveness, and Health: A Review. Front Psychol. 2018;9: 2562 10.3389/fpsyg.2018.02562 30622491 PMC6308207

[4] Thornhill R, Gangestad SW. Human facial beauty—Averageness, symmetry, and parasite resistance. Hum Nat. 1993;4: 237–269. 10.1007/BF02692201 24214366

[5] Thornhill R, Gangestad SW. Facial sexual dimorphism, developmental stability, and susceptibility to disease in men and women. Evol Hum Behav. 2006;27: 131–144. 10.1016/j.evolhumbehav.2005.06.001

[6] Van Dongen S, Gangestad SW. Human fluctuating asymmetry in relation to health and quality: A meta-analysis. Evol Hum Behav. 2011;32: 380–398. 10.1016/j.evolhumbehav.2011.03.002

[7] Zaidi AA, White JD, Mattern BC, Liebowitz CR, Puts DA, Claes P, et al Facial masculinity does not appear to be a condition-dependent male ornament and does not reflect MHC heterozygosity in humans. Proc Natl Acad Sci. 2019; 6 10.1073/pnas.1808659116 PMC6358690 30647112

[8] Lee AJ, Mitchem DG, Wright MJ, Martin NG, Keller MC, Zietsch BP. Facial averageness and genetic quality: testing heritability, genetic correlation with attractiveness, and the paternal age effect. Evol Hum Behav. Elsevier Inc.; 2016;37: 61–66. 10.1016/j.evolhumbehav.2015.08.003 26858521 PMC4743547

[9] Hu B, Shen N, Li JJ, Kang H, Hong J, Fletcher J, et al (2019) Genome-wide association study reveals sex-specific genetic architecture of facial attractiveness. PLoS Genet 15(4): e1007973 10.1371/journal.pgen.1007973 PMC6448826 30946739

[10] Mitchem DG, Purkey AM, Grebe NM, Carey G, Garver-Apgar CE, Bates TC, et al Estimating the Sex-Specific Effects of Genes on Facial Attractiveness and Sexual Dimorphism. Behav Genet. 2014;44: 270–281. 10.1007/s10519-013-9627-5 24213680 PMC4096150

[11] Fink B, Butovskaya M, Sorokowski P, Sorokowska A, Matts PJ. Visual Perception of British Women’s Skin Color Distribution in Two Nonindustrialized Societies, the Maasai and the Tsimane’. Evol Psychol. 2017;15 10.1177/1474704917718957 28727930 PMC10367460

[12] Stephen ID, Scott IML, Coetzee V, Pound N, Perrett DI, Penton-Voak IS. Cross-cultural effects of color, but not morphological masculinity, on perceived attractiveness of men’s faces. Evol Hum Behav. 2012;33: 260–267. 10.1016/j.evolhumbehav.2011.10.003

[13] Wang G, Djafarian K, Egedigwe CA, El Hamdouchi A, Ojiambo R, Ramuth H, et al The relationship of female physical attractiveness to body fatness. PeerJ. 2015;3: e1155 10.7717/peerj.1155 26336638 PMC4556148

[14] Freedman REK, Carter MM, Sbrocco T, Gray JJ. Ethnic differences in preferences for female weight and waist-to-hip ratio: A comparison of African–American and White American college and community samples. Eat Behav. 2004;5: 191–198. 10.1016/j.eatbeh.2004.01.002 15135331

[15] Jones D, Brace CL, Jankowiak W, Laland KN, Musselman LE, Langlois JH, et al Sexual Selection, Physical Attractiveness, and Facial Neoteny: Cross-cultural Evidence and Implications [and Comments and Reply]. Curr Anthropol. 1995;36: 723–748. 10.1086/204427

[16] Muñoz-Reyes JA, Iglesias-Julios M, Pita M, Turiegano E. Facial Features: What Women Perceive as Attractive and What Men Consider Attractive. PLoS ONE. 2015;10 10.1371/journal.pone.0132979 26161954 PMC4498779

[17] Puts DA, Hill AK, Bailey DH, Walker RS, Rendall D, Wheatley JR, et al Sexual selection on male vocal fundamental frequency in humans and other anthropoids. Proc Biol Sci. 2016;283 10.1098/rspb.2015.2830 27122553 PMC4855375

[18] Penton-Voak IS, Jacobson A, Trivers R. Populational differences in attractiveness judgments of male and female faces: Comparing British and Jamaican samples. Evol Hum Behav. 2004;25: 355–370. 10.1016/j.evolhumbehav.2004.06.002

[19] Mansfield E. Too Beautiful to Picture: Zeuxis, Myth, and Mimesis [Internet]. Minneapolis: University of Minnesota Press; 2007 Available: https://muse.jhu.edu/book/32446

